# Problem drug use prevalence estimation revisited: heterogeneity in capture–recapture and the role of external evidence

**DOI:** 10.1111/add.13222

**Published:** 2015-12-28

**Authors:** Hayley E. Jones, Nicky J. Welton, A. E. Ades, Matthias Pierce, Wyn Davies, Barbara Coleman, Tim Millar, Matthew Hickman

**Affiliations:** ^1^ School of Social and Community MedicineUniversity of BristolBristolUK; ^2^ Institute of Brain, Behaviour and Mental HealthUniversity of ManchesterManchesterUK; ^3^ Safer Bristol PartnershipBristol City CouncilBristolUK; ^4^ Public Health Commissioning and PerformanceBristol City CouncilBristolUK

**Keywords:** Hidden population, People Who Inject Drugs (PWID), Bayesian analysis, Bias, Bristol, UK

## Abstract

**Background and Aims:**

Capture–recapture (CRC) analysis is recommended for estimating the prevalence of problem drug use or people who inject drugs (PWID). We aim to demonstrate how naive application of CRC can lead to highly misleading results, and to suggest how the problems might be overcome.

**Methods:**

We present a case study of estimating the prevalence of PWID in Bristol, UK, applying CRC to lists in contact with three services. We assess: (i) sensitivity of results to different versions of the dominant (treatment) list: specifically, to inclusion of non‐incident cases and of those who were referred directly from one of the other services; (ii) the impact of accounting for a novel covariate, housing instability; and (iii) consistency of CRC estimates with drug‐related mortality data. We then incorporate formally the drug‐related mortality data and lower bounds for prevalence alongside the CRC into a single coherent model.

**Results:**

Five of 11 models fitted the full data equally well but generated widely varying prevalence estimates, from 2740 [95% confidence interval (CI) = 2670, 2840] to 6890 (95% CI = 3740, 17680). Results were highly sensitive to inclusion of non‐incident cases, demonstrating the presence of considerable heterogeneity, and were sensitive to a lesser extent to inclusion of direct referrals. A reduced data set including only incident cases and excluding referrals could be fitted by simpler models, and led to much greater consistency in estimates. Accounting for housing stability improved model fit considerably more than did the standard covariates of age and gender. External data provided validation of results and aided model selection, generating a final estimate of the number of PWID in Bristol in 2011 of 2770 [95% credible interval (Cr‐I) = 2570, 3110] or 0.9% (95% Cr‐I = 0.9, 1.0%) of the population aged 15–64 years.

**Conclusions:**

Steps can be taken to reduce bias in capture–recapture analysis, including: careful consideration of data sources, reduction of lists to less heterogeneous subsamples, use of covariates and formal incorporation of external data.

## Introduction

Knowledge of problem drug use prevalence and the number of people who inject drugs (PWID) is critical, serving multiple purposes from estimating the burden of disease locally and globally, informing service planning and evaluating interventions to prevent drug‐related harms 1, 2, 3, 4, 5. Reliable estimates, however, are not straightforward to obtain. Surveys seriously under‐count comparatively rare, stigmatized and vulnerable populations 6, 7, 8, 9, 10, 11. Instead, indirect methods such as capture–recapture (CRC) estimation have been recommended 12, 13, 14, 15. CRC is used extensively to estimate the prevalence of problem drug use 11, 16, 17, 18, 19, 20, 21, 22, 23, 24, 25.

The basic principle of CRC is that prevalence can be estimated based on the pattern of overlap between two, or preferably more, lists of observed individuals from the target population. The number of unobserved individuals is then estimated by fitting a statistical model, usually a log‐linear regression 26, to this overlap. Added to the number observed, this gives the total population size or prevalence. These models are subject to assumptions which, if violated, can lead to invalid estimates 27, 28, 29, 30, 31, 32. Assumptions of independence between data sources (appearing in one list does not affect an individual's likelihood of appearing in another) and homogeneity of ‘capture’ probabilities (all individuals are equally likely to appear in a list) can be relaxed to some extent by incorporating interaction terms and covariates into the regression model 14, 27. However, two key assumptions remain: (1) all variability in capture probabilities is explained by measured covariates; and (2) in an analysis using k lists there is no k‐way dependency 14. It has been noted that overlap between data sources may be inflated by policies to refer between services (e.g. greater integration of criminal justice and drug treatment services) 20. We have demonstrated that even relatively simple referral structures can lead to violations of this second assumption, and consequently to biased results 33. In particular, problems arise if the ‘saturated model’ (which has as many parameters as there are data points) is selected according to conventional model fit criteria, because there are many alternative such models, all of which have perfect fit to the observed data but which can produce widely differing estimates of the population size 14, 33.

In this paper we show, through a case study of estimating the number of PWID in Bristol, UK, how CRC estimates can be highly sensitive to multiple sources of heterogeneity. We demonstrate the need for careful selection of ‘lists’, accounting for routes of referral and use of covariates. In addition, we show how the formal incorporation of other sources of evidence related to PWID prevalence, such as drug‐related deaths, can support the generation of valid estimates 7, 19, 20, 34

## Methods

### Data sources

Our PWID case definition was individuals resident in Bristol aged between 15 and 64 years reporting current or previous injecting and use of opiates and/or crack cocaine during 2011.

Cases were identified from a single database serving multiple service providers (http://www.theseus.org.uk/) comprising: (a) treatment in shared care with primary care or specialist drug treatment services; (b) the Bristol Drugs Project needle and syringe programme (NSP); and (c) the Criminal Justice Intervention Team (CJIT), which undertakes assessments in police custody, probation or prison. Routine linkage of individuals between these sources is undertaken by service providers based on full name (and alternative spellings and known aliases), date of birth and sex.

Individuals in drug treatment were categorized as ‘incident’ (a new treatment onset in 2011 with at least 21 days since last treatment discharge) or ‘non‐incident’ (already in treatment at the beginning of 2011, with no new onset during 2011). Additionally, referrals into treatment were categorized as ‘CJIT’ (referred directly by the CJIT) or ‘non‐CJIT’ [largely general practitioner (GP), non‐statutory drug service or self‐referred].

The following covariates that might influence the ‘capture’ probability (the likelihood of being observed) were available for each of the three data sources: gender, age group (15–24, 25–34 and 35–64 years) and instability of housing (see Supporting information, Appendix for coding of this variable).

### External information: mortality data

We also obtained data on: (i) the number of drug‐related poisoning deaths (DRPs) that occurred in Bristol during 2011, for which opiates and/or crack cocaine were mentioned on the death certificate; and (ii) rates of DRP for a large English cohort of PWID linked to the Office for National Statistics (ONS) mortality register 35, 36. More details are provided in the Supporting information, Appendix. Combined, these data can provide us with an estimate of the number of PWID independently of the CRC.

### Statistical analysis

We undertook the following analytical steps:

- Preliminary analysis (without covariates)
  The overlap data consist of seven observed cell counts representing the number of PWID in each combination of the three sources. We used Poisson log‐linear regression models 26 fitted in Stata version 13.1 to model these observed overlaps. We fitted the eight standard models usually applied in CRC estimation exercises, compared population size estimates, and assessed model fit via the Akaike information criterion (AIC). The most complex of these models accommodates an interaction (dependency) between each pair of lists. This is a ‘saturated’ model, meaning that there are as many parameters as there are data points: it is impossible, therefore, to allow for a three‐way dependency in addition. We compared results from three alternative saturated models in which the three‐way interaction term is included at the expense of one of the two‐way interactions 33.
- The effect of referrals
  We have shown previously that referrals between service providers (e.g. directly from criminal justice into treatment) can produce complex between‐source dependencies, leading potentially to misleading estimates 33. We therefore re‐ran all 11 regression models after excluding cases referred via CJIT from the treatment list.
- The effect of excluding non‐incident cases
  We hypothesized that incident treatment cases might have different capture probabilities (i.e. a greater likelihood of appearing in NSP or CJIT) than those treated for longer periods, as longer‐term treatment might be associated with a less chaotic life‐style. Failure to account for this heterogeneity could bias results. Conversely, if heterogeneity is not present, then estimates should be robust to exclusion of non‐incident cases. We therefore re‐ran all 11 models after restricting the treatment list to incident cases only. We performed this exercise both with and without inclusion of the CJIT referrals.
  The four alternative versions of the treatment list are shown in Box.

**Table:** 

A	Full treatment list
B	CJIT referrals excluded
C	All incident treatment cases
D	Incident treatment cases, excluding CJIT referrals
  Sensitivity of estimates to choice of treatment list was taken as evidence of heterogeneity in the full list. Because this violates the assumptions of CRC, we selected the least heterogeneous treatment list to take forward into the next stages of analysis.
- Incorporation of covariates
  We assumed main effects of, and first‐order interactions between, all three covariates. We then assessed whether model fit improved with the addition of covariate interaction terms. We compared the three models which assumed independence between data sources: (1) no covariates, (2) all three sources depend on gender and age group and (3) all three also depend on housing instability. Thereafter, we considered extensions of the best‐fitting of these to also allow for dependencies between sources.
  These analyses could be performed in any standard statistical software. We used the Bayesian software WinBUGS 37, as this facilitates the extensions in the next analytical step. Bayesian analyses require the specification of ‘prior’ distributions for parameters, quantifying knowledge prior to examination of the data. We assumed vague normal prior distributions for each regression coefficient, representing lack of prior knowledge. The results presented are posterior medians with 95% credible intervals (Cr‐Is). Model fit was compared using the Deviance information criterion (DIC) 38, a Bayesian analogue to the AIC with lower values implying better fit, and posterior mean residual deviance. Well‐fitting models should have mean residual deviance approximately equal to the number of data points (in this case, 84 covariate and source combinations). WinBUGS code for the best‐fitting model is shown in the Supporting information, Appendix.
- Incorporation of external information
  Incorporation of external information alongside a CRC analysis offers potential for model validation or formal identification of inconsistencies. Full details of our incorporation of external information are provided in the Supporting information, Appendix.

Briefly, we first used DRP data in isolation to provide an estimate of the total number of PWID independently from the CRC. The approach used is similar to the ‘multiplier method’ often used for prevalence estimation 8, but with more appropriate accounting for uncertainties.

We then formally incorporated the mortality data alongside the CRC, allowing both sources of information to inform prevalence simultaneously 19, 20, i.e. unifying the two analyses.

Models based on reduced versions of the CRC data (i.e. using treatment lists B–D rather than A) could produce estimates for some subpopulations that are less than the total observed, which would be invalid. We therefore also incorporated formally the total number of observed PWID in each age and gender group as lower bounds.

Because the incorporation of external information has the potential to change the optimal choice of CRC model, we present results from each of the main competing CRC models incorporating the external data. Our final estimates are from the model with the lowest DIC at this stage.

## Results

### Characteristics of the data

The full data, stratified by all covariates and source combinations, are shown in the Supporting information, Appendix 27. In total, 2450 PWID aged 15–64 years were identified: 5% aged 15–24, 38% aged 25–35 and 57% aged 35–64 years. 73% were men and 31% were in ‘unstable’ housing; 285 were in contact with the NSP; 418 were assessed by CJIT; and 2276 (93%) were in treatment during 2011 (full treatment list A).

Removing CJIT referrals reduced the treatment list to 2189 cases (treatment list B) and the full linked data set to 2418. There were 674 incident treatment cases (list C). Inclusion of only these in the treatment list reduced the full data set to 1089. Further removing incident CJIT referrals reduced the treatment list to 573 (list D) and the full linked data set to 1066.

### Statistical analysis

Summary results from analytical stages (i), (ii) and (iii) are displayed in Table 1. Estimated interaction terms from each model are displayed in the Supporting information, Appendix.

- Preliminary analysis
  **Table 1:** Estimated size of the population of people who inject drugs (PWID) in Bristol in 2011: assessment of sensitivity of results to exclusion of CJIT referrals and/or non‐incident cases in the treatment list. Estimated interaction terms from each model are shown in the Appendix, Supporting information.

*Log‐linear model*	*Choice of treatment list (total number of PWID observed in CRC data set)*							
	*A: Full list including CJIT referrals (2450)*		*B: Full list excluding CJIT referrals (2418)*		*C: Incident list including CJIT referrals (1089)*		*D: Incident list excluding CJIT referrals (1066)*	
	*Estimated population size (95% CI)*	*AIC*	*Estimated population size (95% CI)*	*AIC*	*Estimated population size (95% CI)*	*AIC*	*Estimated population size (95% CI)*	*AIC*
(1) No interactions	3080 (2980, 3210)	162	3330 (3190, 3500)	100	1910 (1780, 2070)	90	2350 (2140, 2600)	57
(2) T×N	2740 (2670, 2840)	93	3050 (2920, 3210)	76	1790 (1660, 1950)	84	2380 (2120, 2700)	59
(3) T×C	4000 (3670, 4420)	70	3970 (3640, 4390)	70	2490 (2180, 2890)	58	2470 (2150, 2870)	58
(4) N×C	3100 (2990, 3230)	162	3370 (3220, 3540)	97	1840 (1710, 1990)	86	2320 (2090, 2600)	59
(5) T×N + T×C	4510 (3090, 9150)	72	3750 (3080, 5120)	72	2920 (2230, 4030)	58	2770 (2210, 3620)	58
(6) T×N + N×C	2740 (2670, 2840)	95	3070 (2930, 3240)	76	1680 (1560, 1830)	76	2340 (2060, 2720)	61
(7) T×C + N×C	4260 (3850, 4790)	59	4230 (3810, 4760)	60	2490 (2110, 3010)	60	2460 (2080, 2980)	60
(8) T×N + T×C + N×C	6890 (3740, 17680)	59	5320 (3720, 8900)	60	3540 (2320, 5930)	59	3670 (2350, 6340)	59
(9) T×N + T×C + T×N×C	4510 (3090, 9150)	59	3750 (3080, 5120)	60	2920 (2230, 4030)	59	2770 (2210, 3620)	59
(10) T×N + N×C + T×N×C	2740 (2670, 2840)	59	3070 (2930, 3240)	60	1680 (1560, 1830)	59	2340 (2060, 2720)	59
(11) T×C + N×C + T×N×C	4260 (3850, 4790)	59	4230 (3810, 4760)	60	2490 (2110, 3010)	59	2460 (2080, 2980)	59

T = Treatment, N = needle exchange, C = Criminal Justice Intervention Team; e.g. ‘T×N’ refers to a treatment by needle exchange interaction term in the log‐linear model. AIC = Akaike information criterion. AIC values should be compared within columns only. Estimates from models with the lowest AIC for each data set are shaded. CRC = capture–recapture; CJIT = Criminal Justice Intervention Team; PWID = people who inject drugs; CI = confidence interval.
  Based on the full data set, there was clear evidence from the AIC that at least two interaction terms were required to explain the observed overlap. Evidence for a positive dependence between treatment and the CJIT was particularly strong. Five models had jointly lowest AIC (Table 1, models 7–11). These generated widely varying estimates of the total number of PWID: from 2740 [95% confidence interval (CI) = 2670, 2840] to 6890 (95% CI = 3740, 17680).
- The effect of referrals
  After removing CJIT referrals from the treatment list (list B) there remained evidence for a positive dependence between treatment and the CJIT, but the estimated size of this was reduced considerably. The true dependence structure, however, remained unclear, the same five models still jointly having the lowest AIC. Population size estimates from these models were slightly less variable, but still differed considerably (Table 1).
- The effect of non‐incident cases
  Results were highly sensitive to inclusion of non‐incident treatment cases, providing evidence of variable catchability, i.e. incident and non‐incident treatment cases having different probabilities of appearing in the other lists. Inclusion of only incident cases (treatment lists C and D) reduced the complexity of dependence structures needed to explain the overlap and generated substantially smaller, and more consistent, estimates.
  As there was evidence of heterogeneity in the larger treatment lists (as indicated by sensitivity of results to removal of some cases), we selected the reduced list D to take forward into the next stage. For this data set, the simplest model was preferred based on the AIC, and other models with similar AIC provided similar estimates. We note, however, that the best‐fitting unadjusted (without covariates) model gives an estimate of 2350 (95% CI = 2140, 2600) PWID, which is below the number observed (2450).
- Incorporation of covariates
  Table 2 (models 1–7) displays estimates and model fit statistics from covariate models. Corresponding gender and age group‐specific estimates are shown in Table 3.
  **Table 2:** Estimated size of the injecting population in Bristol in 2011: assessment of sensitivity of results to incorporation of covariates and source dependencies.

*Model*	*Estimated number of PWID (95% Cr‐I)*			*Estimated prevalence (%) of PWID (95% Cr‐I)*			*Model fit*		
	*Male*	*Female*	*Total*	*Male*	*Female*	*Total*	$\overset{‐}{D}$	*pD*	*DIC*
*Covariate capture–recapture models*									
1. No covariates	1850 (1680, 2050)	500 (450, 570)	2350 (2140, 2600)	1.2% (1.1, 1.4)	0.3% (0.3, 0.4)	0.8% (0.7, 0.9)	217	13	230
2. Gender, age	1830 (1650, 2060)	510 (420, 660)	2350 (2140, 2620)	1.2% (1.1, 1.4)	0.3% (0.3, 0.4)	0.8% (0.7, 0.9)	197	22	219
3. Gender, age, housing	2100 (1830, 2470)	580 (460, 780)	2690 (2360, 3140)	1.4% (1.2, 1.6)	0.4% (0.3, 0.5)	0.9% (0.8, 1.1)	98	25	123
*Extensions of model 3 to allow for source dependencies*									
4. T×N	2260 (1890, 2820)	700 (500, 1040)	2970 (2490, 3680)	1.5% (1.2, 1.9)	0.5% (0.3, 0.7)	1.0% (0.8, 1.2)	98	30	127
5. T×C	2400 (1930, 3170)	550 (400, 860)	2960 (2430, 3890)	1.6% (1.3, 2.1)	0.4% (0.3, 0.6)	1.0% (0.8, 1.3)	88	30	117
6. N×C	1910 (1670, 2250)	610 (470, 860)	2530 (2210, 2980)	1.3% (1.1, 1.5)	0.4% (0.3, 0.6)	0.9% (0.7, 1.0)	90	30	120
7. T×C + N×C	2060 (1640, 2950)	590 (400, 1190)	2670 (2140, 3940)	1.4% (1.1, 2.0)	0.4% (0.3, 0.8)	0.9% (0.7, 1.3)	86	35	121
*Capture–recapture subject to constraints and accounting for mortality data* a									
3.^*^ Gender, age, housing^b^	2130 (1920, 2470)	750 (680, 890)	2890 (2650, 3260)	1.4% (1.3, 1.6)	0.5% (0.5, 0.6)	1.0% (0.9, 1.1)	102	23	125
4.^*^ T×N^b^	2200 (1920, 2670)	780 (690, 1010)	3000 (2670, 3520)	1.5% (1.3, 1.8)	0.5% (0.5, 0.7)	1.0% (0.9, 1.2)	97	28	126
5.^*^ T×C^b^	2330 (1990, 2940)	820 (710, 1030)	3170 (2760, 3810)	1.5% (1.3, 1.9)	0.6% (0.5, 0.7)	1.1% (0.9, 1.3)	95	28	123
6.^*^ N×C^b^	2000 (1850, 2290)	760 (680, 930)	2770 (2570, 3110)	1.3% (1.2, 1.5)	0.5% (0.5, 0.6)	0.9% (0.9, 1.0)	91	27	119
7.^*^ T×C + N×C^b^	2080 (1870, 2550)	810 (690, 1090)	2910 (2620, 3490)	1.4% (1.2, 1.7)	0.6% (0.5, 0.7)	1.0% (0.9, 1.2)	91	32	123

a DIC values should not be compared between standard capture–recapture models and those subject to constraints. Estimates shown are posterior medians from Bayesian analyses with 95% credible intervals (Cr‐Is), rounded to the nearest 10. $\bar{D}$ = residual deviance, pD = effective number of parameters, DIC = deviance information criterion (which is calculated as $\bar{D}$ + pD). Models marked with a

b are extensions of the corresponding CRC models to incorporate external data. Final estimates, from the best‐fitting model, are shaded. PWID = people who inject drugs. T = Treatment, N = needle exchange, C = Criminal Justice Intervention Team; e.g. ‘T×N’ refers to a treatment by needle exchange interaction term in the log‐linear model.
  **Table 3:** Estimated size of the injecting population in Bristol in 2011: assessment of sensitivity of results to incorporation of covariates and source dependencies.

*Log‐linear model*	*Men 15–24*	*Men 25–34*	*Men 35–64*	*Women 15–24*	*Women 25–34*	*Women 35–64*
Observed	66	638	1091	54	301	300
*Covariate capture–recapture models*						
1. No covariates	110 (90, 130)	750 (680, 840)	980 (890, 1100)	70 (60, 80)	250 (220, 290)	180 (160, 220)
2. Gender, age	110 (80, 170)	710 (620, 840)	1010 (870, 1180)	70 (50, 120)	240 (200, 320)	190 (150, 260)
3. Gender, age, housing	130 (90, 210)	820 (690, 1010)	1140 (960, 1390)	80 (60, 150)	280 (220, 380)	220 (160, 300)
*Extensions of model 3 to allow for source dependencies*						
4. Treatment × beedle	120 (80, 210)	830 (670, 1080)	1300 (1030, 1710)	90 (50, 180)	320 (230, 490)	280 (190, 450)
5. Treatment × CJIT	250 (130, 670)	970 (740, 1350)	1150 (900, 1540)	120 (60, 330)	250 (180, 390)	170 (120, 250)
6. Needle exchange × CJIT	90 (70, 150)	750 (620, 930)	1060 (890, 1300)	70 (50, 130)	300 (220, 430)	230 (170, 350)
7. Treatment × CJIT + needle x CJIT	190 (90, 870)	840 (620, 1250)	970 (770, 1320)	120 (60, 620)	270 (180, 490)	170 (120, 300)
*Capture–recapture subject to constraints and accounting for mortality data*						
3.^a^ Gender, age, housing	100 (80, 160)	800 (680, 970)	1220 (1100, 1460)	80 (60, 130)	340 (300, 440)	320 (300, 380)
4.^a^ Treatment × needle exchange	100 (70, 160)	790 (670, 1000)	1300 (1110, 1640)	80 (60, 160)	350 (300, 490)	330 (300, 460)
5.^a^ Treatment × CJIT	150 (90, 300)	870 (700, 1170)	1290 (1110, 1670)	130 (70, 240)	360 (300, 510)	320 (300, 390)
6.^a^ Needle exchange × CJIT	90 (70, 120)	740 (650, 900)	1160 (1090, 1370)	70 (60, 120)	350 (300, 470)	320 (300, 410)
7.^a^ Treatment × CJIT + needle x CJIT	100 (70, 250)	780 (650, 1060)	1180 (1090, 1480)	100 (60, 270)	370 (300, 550)	330 (300, 430)
*Prevalence estimates from final selected model*						
Population	35100	39800	76200	35200	37700	73700
Prevalence	0.3% (0.2, 0.3)	1.9% (1.6, 2.3)	1.5% (1.4, 1.8)	0.2% (0.2, 0.3)	0.9% (0.8, 1.2)	0.4% (0.4, 0.6)

Estimates shown are posterior medians from Bayesian analyses with 95% credible intervals, rounded to the nearest 10. Models with an

a are extensions of the corresponding capture–recapture (CRC) model to incorporate external data. Final estimates, from the best‐fitting model, are shaded. CJIT = Criminal Justice Intervention Team.
  Allowing capture probabilities to vary by gender and age group led to some improvement in model fit (11‐point reduction in DIC, relative to baseline model 1), but did not change estimated prevalence. Gender and age group‐specific estimates were also largely robust to incorporation of these covariates, although Cr‐Is became wider.
  Accounting for housing instability led to an additional 96‐point reduction in the DIC, indicating substantially better fit. Incorporation of this covariate led to a slight increase in estimated prevalence for all subgroups (Table 3, model 3) and overall (2690, 95% Cr‐I = 2360, 3140).
  There was evidence for a positive dependence between treatment and the CJIT in younger males (model 5; six‐point reduction in DIC relative to independence model) or for a negative dependence between the NSP and CJIT for men only (model 6; three‐point reduction in DIC). These two models gave estimates of 2960 (95% Cr‐I = 2430, 3890) and 2530 (95% Cr‐I = 2210, 2980), respectively. We also fitted a model in which both types of interaction were accommodated (model 7), but this did not improve the fit further.
- Incorporation of external information
  From the external information, we estimated the rate of DRPs among PWID in Bristol to be 5.7 (95% Cr‐I = 3.9–8.3) per 1000 person‐years. There were 15 such deaths among PWID in Bristol in 2011. This generates an estimate (independent of the CRC) of 2570 PWID (95% Cr‐I = 1330, 4730). Full details are provided in the Supporting information, Appendix.
  Models 3*–7* (extensions of models 3–7, respectively) in Tables 2, 3 show prevalence estimates based on simultaneous modelling of the CRC, mortality data and lower bounds. After incorporating the external data, the preferred model based on the DIC was model 6*. Table 3 shows that model 5 was in conflict with some of the lower bounds, particularly for older women [predicting 170 (95% Cr‐I = 120, 250), whereas there were known to be at least 300]. The degree of conflict between model 6 and the lower bounds was less, resulting in better overall fit when all data were modelled together.
  Our final estimate of the number of PWID in Bristol in 2011 was 2770 (95% Cr‐I = 2570, 3110) or 0.9% (95% Cr‐I = 0.9, 1.0%) of the population aged 15–64 years. PWID prevalence was 1.3% (95% Cr‐I = 1.2, 1.5%) in men and 0.5% (95% Cr‐I = 0.5, 0.6%) in women, and highest among men and women aged 25–34 (1.9 and 0.9%, respectively). We estimate that 89% (95% Cr‐I = 79–95%) of PWID in Bristol were in contact with one of the three local services during 2011, and 82% (95% Cr‐I = 73–88%) were in treatment.
  Our posterior estimate of the DRP rate among PWID in Bristol, after formal incorporation with the CRC data, was 5.6 (95% Cr‐I = 4.1, 7.5) per 1000 person‐years. This reflects some learning about mortality rates from the CRC, in addition to learning about prevalence from the mortality data.

## Discussion

We demonstrate potential biases in CRC estimation, but show that it may be possible to: identify sublists of individuals for whom application of CRC is more likely to be valid; incorporate novel additional covariates; and use external information to test consistency, guide model choice and strengthen the evidence for CRC estimates.

For our case study, standard analysis of the routine data usually used for CRC could not identify a single best estimate: estimates from equally well‐fitting models varied 2.5‐fold. Estimates were sensitive to inclusion of non‐incident treatment cases and deliberate referrals between the data sources. Inclusion of such cases generates considerable heterogeneity that is not accounted for by standard methods.

### Limitations

Despite the progress we have made, the estimates from our case study should be interpreted cautiously. We found evidence of heterogeneity in the full treatment list, violating a key CRC assumption. We therefore reduced this list to a more homogeneous subpopulation before applying CRC techniques. However, CRC assumes that all individuals within covariate groups (whether observed or not) have equal capture probabilities. This means that our CRC‐based estimates relate technically only to those PWID who are similar to those included in the reduced data set. As our target population is, in fact, all PWID, including the longer‐term treated, we then used the total number of observed cases to provide lower bounds. Ideally, we would have accounted instead for heterogeneity in the full list using observed covariates. We explored use of ‘incident’ as a covariate, but these analyses indicated a complex between‐source dependence structure among the non‐incident cases, such that it was not possible to identify a single best‐fitting model. Additional factors for which we were unable to account could also influence capture probabilities 39; for example, crack use, intensity of opiate dependence, pattern of injecting and comorbidity. These were not available from all three data sources.

We considered our target population to be people ‘at risk’ of injecting—encompassing people with a risk of relapse—which is a key quantity for policymakers and studies of injecting risk 2. There is no accepted definition of ‘injecting risk’, with studies showing high rates of relapse after 1 year but reducing substantially after more than 5 years 40, 41. By including all individuals who reported ever injecting, it is conceivable that we have included some who have ceased injecting, which would lead to an overestimate. Ideally we would include only individuals who had injected in the last year, but this information was not recorded consistently and, even if it were, is unlikely to be reliable.

We assumed that all matches were identified correctly, with no misclassification of the target population within data sources. However, unlike many other CRC exercises, we took lists of individuals from a single database in which records are matched on an ongoing basis based on full details (including aliases and potential misspelling), which is likely to minimize misclassification errors.

There are also limitations in our external data. We assume both that the 15 opiate‐ and crack‐related deaths were all PWID (which is never specified on the death certificate) and that the mortality rate generated from the cohort study is accurate (relying upon perfect matching to ONS mortality data). It is conceivable that incorporating other external information could reveal further biases and alter estimates.

### Implications and other evidence

Covariate CRC techniques—where heterogeneity is accommodated within a single model rather than by stratifying data—have been recommended previously, but are not adopted routinely 27, 42. National exercises in England and Scotland 16, 18 involve separate CRC estimation in each of many subgroups. Interaction terms are then unlikely to be statistically significant and will be overlooked. In addition, very few CRC studies in drug misuse have made use of covariates other than gender and age. In our case study, results were more sensitive to incorporation of a variable representing instability of housing (a marker of chaotic life‐style). Similarly, Fisher and colleagues 43 showed that mental health problems were an important factor when estimating prevalence of homelessness. It is important, therefore, to ensure that data sources collect rich information regarding characteristics that might influence ‘catchability’, to aid future prevalence estimation.

The problems around non‐incident treatment cases and referrals between services represent important potential biases to be recognized in future studies. It is our contention that many recent exercises of CRC (including potentially some of our own) may be biased, especially if the estimates have not been validated against other data. The data sets used for national exercises in England 18 include all treated cases (not just incident) and do not account explicitly for referrals 33. The results are therefore subject to the problems we have identified.

Simple ‘multiplier’ methods for estimating prevalence, e.g. based on DRPs, are also subject to bias 4, 8, 44. Techniques essentially using a series or combination of multipliers to minimize bias have been proposed 45, 46, but have not quantified uncertainties or formally assessed consistency of evidence. Incorporation of multiple sources of evidence in a single Bayesian analysis facilitates this, with the potential to also allow for biased evidence 1, 7, 34. Other studies have used a Bayesian approach to incorporate DRP data alongside CRC, but with weaker information on expected mortality rates, such that the impact on prevalence estimates was very low, and without careful assessment of heterogeneity in the CRC 19, 20, 47.

Our prevalence estimate of 0.9% (2770, 95% Cr‐I = 2570, 3110) conflicts with published estimates 18 of PWID prevalence in England at 0.2% (0.2, 0.3%) and in Bristol at 0.5% (1500, 95% CI = 1230, 1760) in 2011, and is towards the lower end of the confidence interval from a 2004 analysis which estimated 1.3% (3280, 95% CI = 1940, 4610) PWID in Bristol 48. However, our new estimate is based on drug‐related mortality data in addition to CRC, providing a stronger evidence base.

## Conclusions

We would like to rescue CRC as a robust and valid method for estimating the prevalence of problem drug use to inform evidence‐based medicine and policy making. Indirect estimation methods are crucial, as household surveys are not a viable alternative. However, there is a need for a step change in the analysis, presentation and justification of CRC in drug misuse. Given the emerging complexity of dependencies and heterogeneity across data sources, researchers may need to re‐focus, working with local experts in order to understand and enrich the available data more effectively, rather than attempting large‐scale national exercises with minimal information.

## Declaration of interests

This study was supported by the Medical Research Council (MRC) Nationally Integrated Quantitative Understanding of Addiction Harms addiction research cluster (grant number G1000021) and the NIHR Health Protection Research Unit in Evaluation of Interventions. T.M. has received research funding from the UK National Treatment Agency for Substance Misuse (now Public Health England) and the Home Office. He is a member of the organizing committee for, and chairs, conferences supported by unrestricted educational grants from Reckitt Reckitt Benckiser/Indivior, Lundbeck, Martindale Pharma and Britannia Pharmaceuticals Ltd, for which he receives no personal remuneration. The views expressed in this paper are those of the authors and not necessarily those of the NHS, the NIHR, Department of Health or Public Health England.

## Supporting information


**Appendix S1** Additional details regarding the statistical methodology, full data set, estimated interaction terms, and code to run the final model in the WinBUGS software.

Supporting info item
