# Supplementary material for: Problem drug use prevalence estimation revisited: heterogeneity in capture–recapture and the role of external evidence
Source: Addiction. 2015 Dec 28;111(3):438–47. doi: 10.1111/add.13222 (PMC4981907; doi:10.1111/add.13222)
Supplement: Supplementary file 1 — Supporting info item [file ADD-111-438-s001.docx]

**Web appendix 1: Coding of ‘instability of housing’ covariate**

We coded housing situation as ‘unstable’ if any of the individual’s records indicated an ‘urgent housing problem’, ‘housing problem’, ‘no fixed abode’ or ‘temporary’ accommodation. Specifically, this encompasses: Direct Access short stay hostel; Night winter shelter; Short term bed and breakfast or other hotel; Sleep on different friend’s floor each night; Squatting; Staying with friends or family as a short term guest; Use night hostels (night-by-night basis); House of Multiple Occupancy; Sleep on streets.

Housing situation was coded as ‘stable’ if all of the individual’s records indicated ‘no housing problem’, or ‘settled’. This encompasses: Approved premises; Local Authority / Registered Social Landlord rented, Own property; Private rented; Settled with friends or family, Supported housing or hostel; Traveller; Caravan.

There was no information on the housing situation of 63 (3%) of individuals: housing was assumed stable for these individuals.

**Web appendix 2: Incorporation of external information**

***Mortality:***

We formally incorporated drug-related mortality data alongside our capture-recapture analysis (1, 2).

In 2011, ONS statistics indicate that there were 18 drug-related poisonings (DRPs, ICD10 codes F11-16; F18-19; X40-44; X60-64; X85; Y10-14) in Bristol. Based on lists of drugs mentioned on each of these death certificates, we classified 15 of the deceased as likely People Who Inject Drugs (PWID). As the number was small, we did not consider stratification of this count by gender and age group.

Independently from this, we obtained information on rates of DRPs from a large cohort of PWID identified through the Drug Data Warehouse (3). This is a large (n=1,000,000) case-linked dataset describing criminal justice and health contacts of substance misusers identified via these sources in England. Records for opiate and/or crack users have been linked to mortality records from the Office for National Statistics (4).

For each Local Authority in England except for Bristol, we obtained from the Drug Data Warehouse the number of DRPs and the corresponding number of person years at risk among People Who Inject Drugs (PWID) identified through contact with treatment services between 2005/06 and 2008/09. Treatment data for Bristol during this time period were unavailable.

In total, 791 DRPs were observed in 179,136 person years. We fitted a random effects meta-analysis model to these data, to estimate the average PWID DRP rate across areas while allowing for between-area variability. The average rate $\xi$was estimated to be 4.8 per 1000 person years (95% CI 4.5-5.2), which we characterised as a Beta(719, 149086) distribution. The between-area standard deviation on the log rate scale was estimated to be $\tau$= 0.23, which we assumed known in the analysis described below. This produces a 95% predictive interval of 3.1 to 7.5 deaths per 1000 person years for the rate in Bristol.

This is consistent with, but more precise than, the prior distribution used for the annual injecting DRP rate by King *et al* (1): based on data from Merrell *et al* (5), they centered their prior distribution around 0.6%, with 90% interval 0.3-1.2%. In addition, we were able to further tailor our prior distribution to be more relevant to Bristol, as follows.

We obtained a small amount of Bristol-specific data on DRP rates. Specifically, in a sample of PWID resident in Bristol in contact with the CJIT between 2005/06 and 2008/09, there were $x=$11 DRPs in 1344 person years, or 8.2 (95% Cr-I 4.2-13.8) deaths per 1000 person years. Analysis in other Local Authorities provided no evidence of mortality rates differing between individuals identified through contact with treatment services or the DIP. This data therefore suggests that the DRP rate in Bristol might potentially be higher than the average across DATs.

Putting these two sources of information together in a simple Bayesian model produces a revised (‘shrunken’) estimate of the DRP rate in Bristol of λ =5.7 deaths per 1000 person years, with 95% credible interval 3.9 to 8.3. Formally, the model producing this estimate of λ is:

$$\begin{matrix} \log(\lambda) \sim N(\log(\xi),\tau^{2}) \\ x\sim Poisson(1344\lambda) \end{matrix}$$

where$\xi$and$\tau$are characterised as above.

A crude estimate of the total number of PWID in Bristol in 2011 is therefore 15/(5.7/1000) = 2632. Our more formal approach was to assume that this number of DRPs was a realisation from a Binomial distribution with order N (the total number of PWID in Bristol) and probability *p = 1-exp(λ).* This functional form for p is based on the assumption of a closed Markov model for PWID with two states over the one year period: alive and DRP. This is an approximation to the truth in which a minority of individuals will have entered or left the risk set during the year. A vague log-normal prior distribution was assumed for N, which was updated to form a posterior distribution based on the observed number of deaths and the information on λ described above. This simple mortality model in isolation produced an estimate of N = 2570 (95% Cr-I 1330 to 4730) PWID in Bristol.

Alternatively, to formally incorporate the mortality data alongside the capture-recapture analysis, the prior distribution for N arises from the capture-recapture log-linear regression model.

***Lower bounds:***

Let us define nknown_g_ as the total number of observed PWID in each gender/age group combination (g=1,…,6), across the full version of the capture-recapture data set (that in which treatment list A is used) and n_g_ as the number used for the final analyses (in which treatment list D is used).

Then a lower bound for the true number of individuals missing from the capture-recapture analysis in each group (say, nmiss_g_) is clearly:

*lbound_g_ = nknown_g_ - n_g_*

These lower bounds were enforced by adding in the assumption that

*lbound_g_ ~ Binomial(nmiss_g_, pnuis_g_)*

where a Uniform prior distribution across the full possible range of 0 to 1 was assumed for each nuisance parameter, *pnuis_g_.*

1. King R., Bird S. M., Overstall A. M., Hay G., Hutchinson S. J. Estimating prevalence of injecting drug users and associated heroin-related death rates in England by using regional data and incorporating prior information, J R Stat Soc a Stat 2014: 177: 209-236.

2. King R., Bird S. M., Hay G., Hutchinson S. J. Estimating current injectors in Scotland and their drug-related death rate by sex, region and age-group via Bayesian capture--recapture methods, Stat Methods Med Res 2009: 18: 341-359.

3. Millar T., Ahmad M., Richardson A., Skodbo S., Donmall M., Jones A. The Drug Data Warehouse: Linking data on drug misusers and drug-misusing offenders. Home Office Research Report, London; 2012.

4. Pierce M., Bird S. M., Hickman M., Millar T. National record linkage study of mortality for a large cohort of opioid users ascertained by drug treatment or criminal justice sources in England, 2005-2009, Drug and alcohol dependence 2015: 146: 17-23.

5. Merrall E. L., Bird S. M., Hutchinson S. J. Mortality of those who attended drug services in Scotland 1996-2006: record-linkage study, The International journal on drug policy 2012: 23: 24-32.

**Web appendix 3: Full data**

| **Gender** | | | **Male** | | | | | | **Female** | | | | | |
| --- | --- | --- | --- | --- | --- | --- | --- | --- | --- | --- | --- | --- | --- | --- |
| **Age group** | | | **15-24 years** | | **25-34 years** | | **35-64 years** | | **15-24 years** | | **25-34 years** | | **35-64 years** | |
| **Housing stable?** | | | **Y** | **N** | **Y** | **N** | **Y** | **N** | **Y** | **N** | **Y** | **N** | **Y** | **N** |
| **T** | **N** | **C** | **Dataset A: Prevalent treatment cases, including referrals from CJIT (full)** | | | | | | | | | | | |
| Yes | Yes | Yes | 1 | 2 | 1 | 14 | 3 | 12 | 0 | 0 | 2 | 7 | 1 | 2 |
| Yes | Yes | No | 2 | 0 | 21 | 21 | 31 | 18 | 1 | 3 | 4 | 7 | 5 | 5 |
| Yes | No | Yes | 6 | 6 | 58 | 58 | 73 | 58 | 4 | 7 | 8 | 19 | 13 | 8 |
| Yes | No | No | 20 | 9 | 291 | 107 | 641 | 195 | 18 | 15 | 183 | 57 | 214 | 45 |
| No | Yes | Yes | 0 | 0 | 0 | 2 | 0 | 1 | 0 | 0 | 0 | 0 | 0 | 0 |
| No | Yes | No | 11 | 6 | 12 | 29 | 28 | 15 | 2 | 4 | 5 | 1 | 2 | 4 |
| No | No | Yes | 2 | 1 | 21 | 3 | 11 | 5 | 0 | 0 | 4 | 4 | 0 | 1 |
| **T** | **N** | **C** | **Dataset B: Prevalent treatment cases, excluding referrals from CJIT** | | | | | | | | | | | |
| Yes | Yes | Yes | 1 | 2 | 1 | 11 | 2 | 10 | 0 | 0 | 2 | 7 | 1 | 2 |
| Yes | Yes | No | 2 | 0 | 21 | 20 | 31 | 18 | 1 | 3 | 4 | 7 | 5 | 5 |
| Yes | No | Yes | 3 | 6 | 50 | 53 | 59 | 50 | 3 | 5 | 8 | 16 | 10 | 7 |
| Yes | No | No | 20 | 8 | 286 | 102 | 630 | 191 | 18 | 14 | 181 | 55 | 214 | 44 |
| No | Yes | Yes | 0 | 0 | 0 | 5 | 1 | 3 | 0 | 0 | 0 | 0 | 0 | 0 |
| No | Yes | No | 11 | 6 | 12 | 30 | 28 | 15 | 2 | 4 | 5 | 1 | 2 | 4 |
| No | No | Yes | 5 | 1 | 29 | 8 | 25 | 13 | 1 | 2 | 4 | 7 | 3 | 2 |
| **T** | **N** | **C** | **Dataset C: Incident treatment cases, including referrals from CJIT** | | | | | | | | | | | |
| Yes | Yes | Yes | 1 | 1 | 0 | 7 | 2 | 8 | 0 | 0 | 1 | 4 | 1 | 2 |
| Yes | Yes | No | 1 | 0 | 7 | 10 | 15 | 11 | 1 | 1 | 1 | 3 | 2 | 4 |
| Yes | No | Yes | 4 | 5 | 21 | 33 | 39 | 28 | 2 | 5 | 1 | 12 | 4 | 3 |
| Yes | No | No | 10 | 4 | 80 | 32 | 161 | 36 | 6 | 4 | 40 | 16 | 35 | 10 |
| No | Yes | Yes | 0 | 1 | 1 | 9 | 1 | 5 | 0 | 0 | 1 | 3 | 0 | 0 |
| No | Yes | No | 12 | 6 | 26 | 40 | 44 | 22 | 2 | 6 | 8 | 5 | 5 | 5 |
| No | No | Yes | 4 | 2 | 58 | 28 | 45 | 35 | 2 | 2 | 11 | 11 | 9 | 6 |
| **T** | **N** | **C** | **Dataset D: Incident treatment cases, excluding referrals from CJIT** | | | | | | | | | | | |
| Yes | Yes | Yes | 1 | 1 | 0 | 4 | 1 | 5 | 0 | 0 | 1 | 3 | 1 | 1 |
| Yes | Yes | No | 1 | 0 | 7 | 9 | 15 | 11 | 1 | 1 | 1 | 3 | 2 | 3 |
| Yes | No | Yes | 1 | 4 | 12 | 24 | 22 | 14 | 1 | 2 | 1 | 6 | 1 | 2 |
| Yes | No | No | 9 | 3 | 73 | 29 | 158 | 34 | 6 | 4 | 39 | 13 | 35 | 8 |
| No | Yes | Yes | 0 | 1 | 1 | 12 | 2 | 8 | 0 | 0 | 1 | 4 | 0 | 1 |
| No | Yes | No | 12 | 6 | 26 | 41 | 44 | 22 | 2 | 6 | 8 | 5 | 5 | 6 |
| No | No | Yes | 7 | 3 | 67 | 37 | 62 | 49 | 3 | 5 | 11 | 17 | 12 | 7 |

T = Treatment, N = Needle and Syringe Program (NSP), C = Criminal Justice Intervention Team (CJIT)

An additional 4 injectors were identified, but were excluded from our analyses due to being aged 65 years or older.

**Web appendix 4:** Estimated interaction terms in Poisson log linear models (Table 1 of paper): rate ratios with 95% CIs. Bold font indicates p-value < 0.05.

| Model | Interaction | **A: Full list including CJIT referrals** | **B: Full list excluding CJIT referrals** | **C: Incident list including CJIT referrals** | **D: Incident list excluding CJIT referrals** |
| --- | --- | --- | --- | --- | --- |
| 2 | Treatment x Needle | **0.2 (0.1, 0.3)** | **0.4 (0.3, 0.6)** | **0.6 (0.5, 0.9)** | 1.1 (0.8, 1.5) |
| 3 | Treatment x CJIT | **5.8 (4.0, 8.3)** | **2.5 (1.8, 3.5)** | **2.5 (1.9, 3.5)** | 1.2 (0.9, 1.7) |
| 4 | Needle x CJIT | 1.3 (1.0, 1.9) | **1.5 (1.1, 2.1)** | **0.6 (0.5, 0.9)** | 0.9 (0.6, 1.3) |
| 5 | Treatment x Needle  Treatment x CJIT | 1.3 (0.4, 4.3)  **7.5 (2.4, 23.8)** | 0.9 (0.4, 1.7)  **2.2 (1.1, 4.4)** | 1.4 (0.9, 2.4)  **3.2 (2.0, 5.2)** | 1.3 (0.9, 2.1)  1.4 (0.9, 2.2) |
| 6 | Treatment x Needle  Needle x CJIT | **0.2 (0.2, 0.3)**  1.1 (0.8, 1.6) | **0.5 (0.3, 0.6)**  1.3 (0.9, 1.8) | **0.6 (0.4, 0.8)**  **0.6 (0.4, 0.8)** | 1.1 (0.7, 1.5)  0.9 (0.6, 1.4) |
| 7 | Treatment x CJIT  Needle x CJIT | **6.7 (4.5, 9.7)**  **2.0 (1.4, 2.8)** | **2.9 (2.1, 4.1)**  **2.0 (1.4, 2.8)** | **2.5 (1.8, 3.6)**  1.0 (0.7, 1.5) | 1.2 (0.8, 1.7)  1.0 (0.7, 1.5) |
| 8 | Treatment x Needle  Treatment x CJIT  Needle x CJIT | 2.5 (0.7, 8.2)  **15.1 (4.6, 50.0)**  **2.2 (1.5, 3.1)** | 1.6 (0.8, 3.4)  **4.4 (2.1, 9.6)**  **2.2 (1.5, 3.2)** | 1.7 (1.0, 3.2)  **4.2 (2.2, 7.9)**  1.3 (0.8, 2.2) | 1.9 (1.0, 3.5)  **2.0 (1.1, 3.9)**  1.5 (0.9, 2.7) |
| 9 | Treatment x Needle  Treatment x CJIT  3-way dependency | 1.1 (0.4, 3.7)  **7.0 (2.2, 22.2)**  **2.2 (1.5, 3.1)** | 0.7 (0.4, 1.5)  **2.0 (1.0, 4.1)**  **2.2 (1.5, 3.2)** | 1.3 (0.8, 2.2)  **3.1 (1.9, 5.1)**  1.3 (0.8, 2.2) | 1.2 (0.8, 2.0)  1.3 (0.9, 2.1)  **1.5 (0.9, 2.7)** |
| 10 | Treatment x Needle  Needle x CJIT  3-way dependency | **0.2 (0.1, 0.2)**  **0.1 (0.0, 0.5)**  **15.1 (4.6, 50.0)** | **0.4 (0.3, 0.5)**  **0.5 (0.2, 1.0)**  **4.4 (2.1, 9.6)** | **0.4 (0.3, 0.6)**  **0.3 (0.2, 0.5)**  **4.2 (2.2, 7.9)** | 0.9 (0.6, 1.3)  0.7 (0.5, 1.2)  **2.0 (1.1, 3.9)** |
| 11 | Treatment x CJIT  Needle x CJIT  3-way dependency | **6.2 (4.2, 9.1)**  0.9 (0.3, 2.9)  2.5 (0.7, 8.2) | **2.8 (2.0, 3.9)**  1.4 (0.7, 2.8)  1.6 (0.8, 3.4) | **2.4 (1.7, 3.4)**  0.8 (0.5, 1.3)  1.7 (1.0, 3.2) | 1.1 (0.7, 1.6)  0.8 (0.5, 1.3)  1.9 (1.0, 3.5) |

**Web appendix 5: WinBUGS code for the full model, incorporating mortality data and lower bounds**

model{

for(i in 1:groups){

for(j in 1:cells){

n[i,j] ~ dpois(lambda[i,j])

resdev.crc[i,j] <- 2*( (lambda[i,j]-n[i,j]) + n[i,j]*log(n[i,j]/lambda[i,j]) )

log(lambda[i,j]) <- log(nmiss[i]) +

treat[j]*(t.alpha[1] + t.alpha[2]*M[i] + t.alpha[3]*A2[i] + t.alpha[4]*A3[i] + t.alpha[5]*H[i]) +

needle[j]*(n.alpha[1] + n.alpha[2]*M[i] + n.alpha[3]*A2[i] + n.alpha[4]*A3[i] + n.alpha[5]*H[i]) +

cjit[j]*(c.alpha[1] + c.alpha[2]*M[i] + c.alpha[3]*A2[i] + c.alpha[4]*A3[i] + c.alpha[5]*H[i]) +

needle[j]*cjit[j]*(int.alpha[1] + int.alpha[2]*M[i] + int.alpha[3]*A2[i] + int.alpha[4]*A3[i] + int.alpha[5]*H[i])

}

}

dev.crc <- sum(resdev.crc[,]) # Residual deviance

# Vague priors for all regression coefficients:

for(k in 1:10){

alpha[k] ~ dnorm(0, 0.001)

}

for(k in 1:5){

t.alpha[k] ~ dnorm(0, 0.001)

n.alpha[k] ~ dnorm(0, 0.001)

c.alpha[k] ~ dnorm(0, 0.001)

int.alpha[k] ~ dnorm(0, 0.001)

}

for(i in 1:groups){

totn[i] <- sum(n[i,])

log(nmiss[i]) <- alpha[1] + alpha[2]*M[i] + alpha[3]*A2[i] + alpha[4]*A3[i] + alpha[5]*H[i] + alpha[6]*M[i]*A2[i] + alpha[7]*M[i]*A3[i]+ alpha[8]*H[i]*A2[i] + alpha[9]*H[i]*A3[i] + alpha[10]*M[i]*H[i]

}

for(k in 1:6){

nm[k] <- sum(nmiss[((2*k)-1):(2*k)])

nobs[k] <- sum(totn[((2*k)-1):(2*k)])

Ngroup[k] <- nobs[k] + nm[k] # Numbers of PWID for each gender/age group combination

lbound[k] ~ dbin(pnuis[k], nm[k]) # Lower bounds on missing PWID

pnuis[k] ~ dunif(0,1)

}

Ntot <- sum(Ngroup[])

mean.rate ~ dbeta(719.0617, 149085.5) # Average DRP rate across areas, from external meta-analysis

mu <- log(mean.rate)

bris.mort.log ~ dnorm(mu, 19.63633) # Predictive distribution for DRP rate in new area (log scale)

log(bris.mort.rate) <- bris.mort.log

deaths.past <- 11 # Historic Bristol-specific DRP data

mean.past <- bris.mort.rate*1344

deaths.past ~ dpois(mean.past)

deaths2011 <- 15 # Relevant Bristol DRPs in this year

deaths2011 ~ dbin(prob.death, Ntot)

prob.death <- 1 - exp(-bris.mort.rate)

}

# DATA 1:

M[] A2[] A3[] H[] n[,1] n[,2] n[,3] n[,4] n[,5] n[,6] n[,7]

1 0 0 0 1 1 1 9 0 12 7

1 0 0 1 1 0 4 3 1 6 3

1 1 0 0 0 7 12 73 1 26 67

1 1 0 1 4 9 24 29 12 41 37

1 0 1 0 1 15 22 158 2 44 62

1 0 1 1 5 11 14 34 8 22 49

0 0 0 0 0 1 1 6 0 2 3

0 0 0 1 0 1 2 4 0 6 5

0 1 0 0 1 1 1 39 1 8 11

0 1 0 1 3 3 6 13 4 5 17

0 0 1 0 1 2 1 35 0 5 12

0 0 1 1 1 3 2 8 1 6 7

END

# DATA 2:

list(

groups = 12,

cells = 7,

treat = c(1,1,1,1,0,0,0),

needle = c(1,1,0,0,1,1,0),

cjit = c(1,0,1,0,1,0,1),

lbound = c(17, 296, 644, 23, 188, 216)

)
